# Editorial: New Horizons in Health-Promoting Technologies: From Development to Rational Use

**DOI:** 10.3389/fphar.2020.01180

**Published:** 2020-08-06

**Authors:** Luciane Cruz Lopes, Brian Godman, Cristiane De Cássia Bergamaschi, Silvio Barberato-Filho, Marcus Tolentino Silva

**Affiliations:** ^1^ Pharmaceutical Science Graduate Course, University of Sorocaba, Sorocaba, Brazil; ^2^ Karolinska University Hospital Huddinge, Karolinska Institutet (KI), Stockholm, Sweden; ^3^ Strathclyde Institute of Pharmacy and Biomedical Sciences, University of Strathclyde, Glasgow, United Kingdom; ^4^ School of Pharmacy, Sefako Makgatho Health Sciences University, Garankuwa, South Africa

**Keywords:** health technology assessment, new horizon, rational use of medicines, implementation, drug utilization research

This Research Topic covers 30 articles focusing on recent advancements related to the balance in innovation technology and the rational use of medicine for better decision making.

Independent drug information comes from publications with no conflicts of interest, performed by transparent and robust methods that validate the results that can subsequently be used to improve health outcomes. The publication of studies of high methodological quality helps to identify evidence about the benefits of medicines and technologies that are widely used as well as for those that show substantial variation in their use but without improvement in health outcomes. No less important are the findings that demonstrate which technologies that have proven to be ineffective or have harmful effects, which continue to be used often driven by hype, hope, lack of national guidance, or other pressures including financial pressures. We have seen this with the considerable hype and misinformation surrounding the use of hydroxychloroquine in the treatment of patients with COVID-19.

In a current scenario of limited financial resources, health managers are under pressure, which requires greater efficiency in the use of public money. They need consistent and substantiated information about the benefits and harm of health technologies and their impact on patients and health services to guide future decision making. This Research Topic points out studies that can contribute to this theme, having as a main focus the adequate and rational use of a medicine and other technologies. Rational use of medicine occurs when patients receive the appropriate medicines in doses that meet their own individual requirements, for an adequate period of time, and at the lowest cost both to them and their community (WHO, 2004).

Inappropriate use of medicines can be costly and extremely harmful (related illness and deaths), both to the individual and the population as a whole (Holloway and Van Dijk, 2011). Few healthcare systems presently control the inappropriate use of medicines, which in some is due to a lack of consciousness of the size of the problem and its economic and health burden. In others, decision-makers often lack knowledge of the most cost-effective ways to manage this important problem (Lima et al., 2017) or have a reluctance to instigate demand-side measures to improve appropriate use through pressures from key stakeholder groups (Lopes et al., 2010). Particularly, challenges and barriers to be overcome in low- and middle-income countries are still considerable (Salas et al., 2020).

Problems related to shortages, especially considering the global context of those considered essential, is a topic that can contribute to the irrational use of medicines. Acosta et al. address this issue through a scoping review in which the authors identified an appreciable number of countries that are introducing legislative actions to address shortages of medicines and discuss interest in international cooperation for their prevention and ways to facilitate actions that provide a timely response.

The responsible use of medicines implies that existing activities, capacities, resources, and key stakeholders are aligned to ensure the rational use of medicines (WHO, 2012). Rational use, including issues of adherence to medicines, is enhanced by the instigation of universal healthcare including the continual availability of a key list of essential medicines (Yamauti et al., 2015; Araujo et al., 2016; Yamauti et al., 2017). This is important particularly in low-income countries where the cost of medicines can account for up to 60% or so of total healthcare expenditure, much of which is out of pocket (Cameron et al., 2009) and should be addressed as part of Sustainable Development Goals.

Interventions, including effective drug monitoring and regulations, and encouraging rational use, have been well described in studies that have addressed, among others, the translation of knowledge through a brief political and deliberative discourse (de Araújo et al.; Fulone et al.). In addition, aspects related to the challenges and impact of control policies were addressed in the study by Ranabhat et al. Some authors have shown that when there is a lack of effective drug control and monitoring, either due to wrong selection of essential drugs for reimbursement or lack of demand-side measures (Godman et al., 2014; Fulone et al., 2016; Osorio-De-Castro et al., 2018) or lack of adherence of official guidelines, health and expenditures (Silveira et al., 2014; De Camargo et al., 2016) may be compromised.

It is worth mentioning that in this scenario, in addition to translating knowledge and control policies (Wettermark et al., 2009), the participation of health professionals in a responsible way to expand access and the responsible use of the medicines, mainly in the vulnerable population, assumes significant relevance (Soler and Barreto; Silva et al.). Observational studies carried out with health professionals showed that clinical practice also needs to be reviewed and improved, suggesting significant gaps in the knowledge of appropriate prescriptions (Benko et al.; Fadare et al.).

A good decision should include society’s values, the interests of those potentially involved, and an appreciation of local policies. There is widespread recognition of the need to use information more effectively to inform public health policies, programs, and administrative decisions. To consistently contribute to this theme, some authors of this Research Topic have also included studies that provided important tools to improve health decision-making (Ali et al.; Barcelos et al.) to assist in clinical practice (Félix et al.; Motter et al.; Amodeo et al.) and the quality of the methods used in primary studies (Ali et al.).

New technologies are registered based on controlled and randomized clinical trials, in which, in most cases, they fail to capture important safety results that only appear in real-life studies (Lopes et al., 2014; Fulone et al., 2018; De Carmago et al., 2019). In addition, in the case of new cancer medicines, often licensed on the basis of limited information, which can cause concern and wasted resources (Pontes et al., 2020).

This Research Topic brings together interesting features specified for secondary (Andrade et al.; Lee et al.; Liu et al.; Mellone et al.; Mezones-Holguin et al.) and primary studies (Cavalcanti et al.; Gomes et al.) of technologies already used in several countries. However, doubts related to their effectiveness and safety still point out that they need to be better studied in different contexts. Part of the continued growth in health spending is attributable to the increasing production of new technologies and changes in the population’s epidemiological profile. Studies that indicate whether a technology is cost-effective contribute to adjustments in health policies adopted. The cost-effectiveness study developed by Vecoso et al. showed that the chemoprophylaxis of influenza A (H1N1) is cost-saving in the Brazilian health system context. On the other hand, a number of Brazilian studies have cast doubt on the inclusion of insulin glargine within public health systems in Brazil given appreciable higher prices that existed versus NPH insulins (Caires De Souza et al., 2014; Marra et al., 2016).

In addition, the rapid emergence of high-priced innovations is another major challenge faced by decision-makers. In order to respond, the identification of future innovations and trends should be carried out in a comprehensive, systematic, and sustainable manner so that policymakers and other interested parties can respond appropriately and improve the managed entry of new medicines starting with horizon scanning and budgeting activities, followed by funding and reimbursement decisions and subsequently post launch patient-level studies (Malmström et al., 2013; Godman et al., 2015). The comprehensive model in Sweden provides an exemplar to others (Eriksson et al., 2017; Eriksson et al., 2019). Authors have also discussed important aspects related to gaps in gene therapy assessment (Jolly et al.) and new technologies to combat multidrug-resistant bacteria (Lima et al.).

To improve the input from new developing technologies, this Research Topic also brought together interesting studies that show preliminary experiments, in “*in vitro*” phase, using, among others, biopharmaceutical techniques that modify the pharmaceutical form currently commercialized to increase the permeation of molecules in the skin (Vigato et al.) or through the encapsulation of antibiotics that result in increased antimicrobial effectiveness (Scriboni et al.) addressing concerns with the lack of new antibiotics being developed to address rising resistance concerns. In addition, discussions concerning combined delivery systems based on nanostructures to administer drugs by the oral cavity (Feitosa et al.). Tests with promising agents to treat diabetes (Muñoz-Talavera et al.) or osteoporosis have also been presented (Hou et al.). We will be following up their development.

## Author Contributions

LL designed conception and wrote the draft of editorial. BG, CB, SB-F, and MS made substantial contributions, revising it critically for important intellectual content. All authors contributed to the article and approved the submitted version.

## Conflict of Interest

The authors declare that the research was conducted in the absence of any commercial or financial relationships that could be construed as a potential conflict of interest.

The handling editor declared a past co-authorship with one of the authors BG.
